# *Aronia melanocarpa* (Michx.) Elliot, *Chaenomeles superba* Lindl. and *Cornus mas* L. Leaf Extracts as Natural Preservatives for Pork Meat Products

**DOI:** 10.3390/molecules26103009

**Published:** 2021-05-18

**Authors:** Magdalena Efenberger-Szmechtyk, Ilona Gałązka-Czarnecka, Anna Otlewska, Agata Czyżowska, Agnieszka Nowak

**Affiliations:** 1 Institute of Fermentation Technology and Microbiology, Lodz University of Technology, Wolczanska 171/173, 90-924 Lodz, Poland; anna.otlewska@p.lodz.pl (A.O.); agata.czyzowska@p.lodz.pl (A.C.); agnieszka.nowak@p.lodz.pl (A.N.); 2 Institute of Food Technology and Analysis, Lodz University of Technology, Stefanowskiego 4/10, 90-924 Lodz, Poland; ilona.galazka-czarnecka@p.lodz.pl

**Keywords:** *Aronia melanocarpa*, *Chaenomeles superba*, *Cornus mas*, meat, 16S rRNA gene sequencing, natural preservatives

## Abstract

The aim of this study was to investigate the possibility of using *Aronia melanocarpa*, *Chaenomeles* *superba*, and *Cornus mas* leaf extracts as natural preservatives for pork meat products. Pork sausages were stored in modified atmosphere packaging (MAP) (80% N_2_ and 20% CO_2_) at 4 °C for 29 days. The total psychrotrophic counts (TPC) were determined during the storage period, along with the numbers of *Enterobacteriaceae* and lactic acid bacteria (LAB). The extracts improved the microbial quality of the meat products but to a lesser extent than sodium nitrate (III). They reduced the amounts of *Enterobacteriaceae* and LAB. The *A.* *melanocarpa* leaf extract showed the strongest preservative effect. The bacterial biodiversity of the meat products was investigated based on high-throughput sequencing of the 16S rRNA gene. Two predominant bacteria phyla were identified, *Proteobacteria* and *Firmucutes*, mostly consisting of genera *Photobacterium,* *Brochothrix*, and *Carnobacterium*. The extracts also influenced microbial community in sausages decreasing or increasing bacterial relative abundance. The extracts significantly inhibited lipid oxidation and improved the water-holding capacity of the meat, with *C. superba* extract showing the strongest influence. In addition, *A. melanocarpa* and *C. superba* improved the redness (a*) of the sausages. The results of this study show that *A. melanocarpa*, *C. superba,* and *C. mas* leaf extracts can extend the shelf life of meat products stored in MAP at 4 °C.

## 1. Introduction

Meat is a good environment for the growth of many microorganisms. The following bacteria are commonly found in meat and meat products: *Pseudomonas fragi, Pseudomonas fluorescens, Acinetobacter* sp., *Moraxella* sp., *Escherichia coli, Staphylococcus aureus, Flavobacterium* sp., *Micrococcus* sp., *Bacillus* sp., *Streptococcus* sp., *Serratia* sp., *Carnobacterium* sp., *Lactobacillus* sp., *Lactococcus* sp., *Leuconostoc* sp., *Brochothrix thermosphacta*, *Enteroccus* sp., *Proteus* sp., *Psychrobacter* sp., *Alkaliphilus* sp., *Kluyvera* sp. and *Clostridium* sp. Fungi such as *Candida* sp., *Rhodotorula* sp., *Cladosporium* sp., and *Cryptococcus* sp. can also occur, but they are not predominant microflora. Meat products can also become contaminated with pathogenic bacteria, such as *Listeria monocytogenes*, *Salmonella* sp., *Clostridium botulinum*, *Yersinia enterocolitica, Campylobacter jejuni,* and pathogenic strains of *E. coli* [1,2,3,4].

To inhibit the growth of microorganisms and extend the shelf life of meat products, food preservatives are routinely added during meat processing [5,6]. Using food preservatives is regulated by Commission Regulation (EC) No 1333/2008 of the European Parliament and of the Council of 16 December 2008 on food additives. Preservatives permitted to be used in meat and meat products include sulfur dioxide-sulfates (IV) (E220–228), potassium nitrate (III) (E249), sodium nitrate (III) (E250), sodium nitrate (V) (E251), potassium nitrate (V) (E252), acetic acid (E260), potassium acetate (E261), sodium acetate (E262), calcium acetate (E263), sorbic acid-sorbates (E200–203), benzoic acid-benzoates (E210–213), p-hydroxybenzoates (E214-219), natamycin (E235), and lactic acid (E270). In cured pork sausages, which were prepared in our studies, nitrates (III) (E249 and E250) are permitted to be used. Nitrates (III), usually in mixtures with salt, have many useful properties. First of all, they inhibit the growth of spoilage and pathogenic bacteria (including spore-forming *Clostridium botulinum*). They also reduce the oxidation of meat ingredients and improve the organoleptic properties of meat. Finally, they give to meat products their characteristic pink-red color. However, nitrates (III) can be very harmful to human health. Heating processes (t > 130 °C), such as grilling or frying, and near acidic pH support the reaction of nitrates (III) with amino compounds in meat, leading to the formation of highly carcinogenic nitrosamines [7,8].

There is great interest in finding new and natural methods of meat preservation, which would be safe for human consumption and improve the health benefits of food products. Due to their antioxidant and antibacterial properties, plant extracts rich in polyphenols can effectively inhibit meat spoilage processes and thus offer a promising alternative to chemical preservatives [9,10,11]. Moreover, polyphenols have well-documented health benefits, such as anti-diabetic, anti-allergic, anti-atherogenic, anti-hypertensive, anti-thrombotic, cardioprotective, osteoprotective, neuroprotective, anti-aging, hepatoprotective, and anti-cancer effects [12]. Studies have shown that plant extracts containing high amounts of polyphenols effectively inhibit the growth of spoilage and pathogenic microflora in meat, oxidation processes, discoloration, and organoleptic changes [9,11].

In recent years, there has been a growing interest in the application of plant leaf extracts rich in polyphenols to meat and meat products, including olive [13], blackcurrant and sour cherry leaf extracts [14], cork oak [15], bamboo [16], eucalyptus [17], *Moringa oleifera* and *Bidens pilosa* leaf extracts [18], and curry and mint [19] leaf extracts. According to the literature, the leaves contain larger amounts of polyphenols than other parts of plants [20,21]. Unlike fruits, leaves do not contain simple sugars, which can stimulate the growth of microorganisms. Leaves are usually considered a waste material. When they fall from trees, they may be used to protect plants during the winter or in the production of compost. Due to the exceptional properties, abundance, and low cost of leaves, there is great potential for the use of leaf extracts as natural preservatives in the meat industry.

Apart from polyphenolic extracts, essential oils also seem to be promising natural preservatives for meat and meat products. Šojić et al. [22] documented the use of wild-thyme by-products in ground pork patties. The extracts protected from color degradation, lipid, and protein oxidation as well as reduced microbial counts in meat products. Stojanović-Radić et al. [23] reported that basil and rosemary essential oils exerted antioxidant effect in chicken meat and reduced the amounts of pathogenic bacteria *Salmonella* Enteritidis. 

Our previous studies showed that *A. melanocarpa, C. superba*, and *C. mas* leaf extracts are rich sources of bioactive compounds—mainly polyphenols (phenolic acids and flavonols) with strong antioxidant properties. In addition, *C. superba* extract was found to contain flavones and flavanones, while ellagitannins and iridoids (non-phenolic antioxidant compounds) were identified in the *C. mas* extracts. The extracts showed antibacterial activity against meat spoilage and pathogenic bacteria [24,25]. We further demonstrated that *A. melanocarpa, C. superba,* and *C. mas* leaf extracts possess cytotoxic and genotoxic properties toward the human colon adenocarcinoma cell line Caco-2, which may indicate anticancer activity [26]. To our knowledge, there have been no previous studies in which extracts from the leaves of *A. melanocarpa, C. superba,* and *C. mas* were applied to meat and meat products.

The aim of the present study was to investigate the application of *A. melanocarpa, C. superba,* and *C. mas* leaf extracts as natural preservatives in pork sausages, as alternatives to nitrates (III). We evaluated the influence of the leaf extracts on the microbial stability, lipid oxidation, drip loss, color parameters, and sensory properties of pork sausages stored at 4 °C in MAP. We also investigated the effects of the extracts on the microbial diversity of the meat products, which was based on high-throughput sequencing of the 16S rRNA gene.

## 2. Results and Discussion

### 2.1. Effect of Leaf Extracts on Microbial Counts in Meat Products

In our previous studies, we demonstrated that *A. melanocarpa, C. superba,* and *C. mas* extracts show antibacterial activity against typical meat spoilage and pathogenic bacteria. The leaf extracts decreased the bacterial growth rate (µ_max_) and extended the lag phase (t_Lag_), indicating a bacteriostatic effect [24]. In further studies, the leaf extracts showed bactericidal activity and decreased bacteria viability [25]. Therefore, in the current research, we investigated the application of *A. melanocarpa, C. superba,* and *C. mas* leaf extracts as natural preservatives in pork meat products. We evaluated if they could be alternatives to nitrates (III), which are commonly used preservatives in meat products that are harmful to human health. The influence of *A. melanocarpa, C. superba,* and *C. mas* leaf extracts on the shelf life of pork sausages was investigated during 29 days of refrigerated storage in MAP. We prepared the following variants of sausages: AMS—sausage with *A. melanocarpa* leaf extract (5% *v*/*w*); ChS—sausage with *C. superba* leaf extract (5% *v*/*w*); CMS—sausage with *C. mas* leaf extract (5% *v*/*w*). We also prepared sausages containing sodium nitrate (III)—NS sample. An SS sample without plant extract and nitrates (III) was a reference sample in this study. 

Curing salt containing sodium nitrate (III) had the strongest influence on the microbial stability of the meat products during storage at 4 °C. The greatest increase in the number of bacteria was noted in the SS sample. Generally, the extracts inhibited the growth of microorganisms in the sausages (compared to SS). The preservative effect depended on the type of extract, storage time, and the group of microorganisms (Table 1). 

Among the extracts studied, A. *melanocarpa* had the strongest effect on the microbial parameters of the meat products. (Table 1). The extract had an inhibitory effect on all the studied groups of microorganisms, over the whole storage period, but at varying degrees. *A. melanocarpa* extract was the most active toward *Enterobacteriaceae.* Similarly to sodium nitrate (III), the extract significantly reduced *Enterobacteriaceae* counts up to 15 days of storage compared to the SS sample. After that time, the extract still had antibacterial activity, although the influence was lower. *C superba* also inhibited *Enterobacteriaceae* up to 15 days, although to a lesser degree. The *C. mas* extract showed an inhibitory effect on *Enterobacteriaceae* at the beginning of storage (up to 8 days).

Regarding lactic acid bacteria (LAB) counts, *A. melanocarpa* inhibited its growth significantly up to 15 days of storage. Interestingly, the *C. mas* extract did not influence the results for LAB at the beginning of the storage period (up to day 4). However, after 8–15 days of storage, the *C. mas* extract reduced LAB compared to SS. It is worth noting that after 11 and 15 days of storage, LAB counts in AMS and CMS samples were lower than in the NS sample. *C. superba* inhibited LAB up to 8 days of storage.

The extracts showed weak antibacterial activity toward psychrotrophic bacteria. Only sodium nitrate (III) strongly reduced total psychrotrophic count (TPC). *A. melanocarpa* and *C. mas* extracts decreased TPC in a statistically significant manner (*p* < 0.05), but from a practical point of view, it was irrelevant.

Therefore, *A. melanocarpa, C. mas,* and *C. superba* leaf extracts showed an inhibitory effect on *Enterabacteriaceae* and LAB. The extracts improved the microbial quality of meat products up to 15 days of storage. The extracts revealed slightly weaker activity than sodium nitrate (III); however, they sufficiently inhibited the growth of microorganisms and can replace the use of nitrates (III). No significant effect of leaf extracts on TPC can result from differences in bacterial communities, as evidenced further by metagenomics analysis.

**Figure 1:**
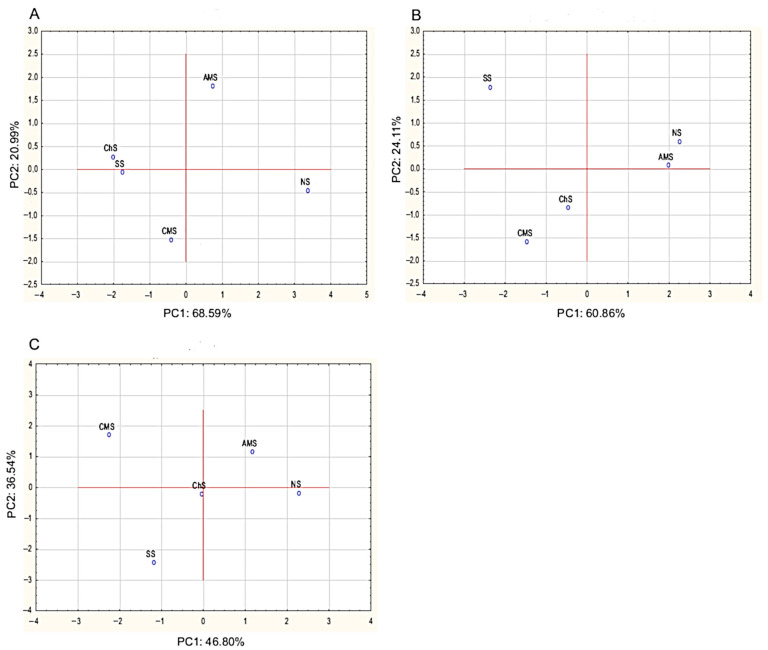
Principal Component Analysis (PCA) showing the effects of *Aronia melanocarpa, Chaenomeles superba,* and *Cornus mas* leaf extracts on the microbial quality of pork sausages during 29 days of refrigerated storage in MAP. AMS—sausage with *Aronia melanocarpa* leaf extract (5% *v*/*w*); ChS—sausage with *Chaenomeles superba* leaf extract (5% *v*/*w*); CMS—sausage with *Cornus mas* leaf extract (5% *v*/*w*); NS—sausage with curing salt containing sodium nitrate (III); SS—control sausage with salt and spices only (without extract and nitrates (III)); (**A**)—total psychrotrophic count (TPC); (**B**)—*Enterobacteriaceae*; (**C**)—lactic acid bacteria (LAB).

Figure 1 presents a Principal Component Analysis (PCA) of the influence of *A.* *melanocarpa, C. superba,* and *C. mas* leaf extracts on the population of bacteria (TPC, *Enterobacteriaceae*, LAB) in the pork sausages. PC1 and PC2 explain 89.58% of total variance for TPC, 84.97% for *Enterobacteriaceae* and 83.34% for LAB. PCA confirms that sodium nitrate (III) as well as *A. melanocarpa, C. superba,* and *C. mas* leaf extracts show antibacterial activity in meat. In Figure 1A, the ChS sample is located close to the SS sample, indicating that the *C. superba* extract had no effect on TPC, whereas the NS, AMS, and CMS samples are separated from the SS sample, suggesting inhibitory activity. In the case of *Enterobacteriaceae* and LAB (Figure 1B,C), the samples are located far from the SS sample, which suggests that all the extracts inhibited the growth of these bacteria groups.

Our previous studies had found that *C. mas* extract had the strongest antibacterial activity against bacteria strains typical for meat, followed by *C. superba* and *A. melanocarpa* [24,25]. However, in the present study, we found that *A. melanocarpa* increased the shelf life of the meat products the most. This may be associated with the different absorption of bioactive compounds in the extracts in the meat environment. It has been reported that polyphenols can interact with food components such as proteins, carbohydrates, fiber, and fat, which can affect their absorption and also change their bioactivity [27,28].

Saleh et al. [13] investigated the effect of olive leaf extract on the microbial quality of poultry meat. The extract reduced the total mesophilic count (TMC), TPC, total *Enterobacteriaceae* count, total staphylococcal count, and total mold and yeast counts, compared to the control. The extract had less of an influence on the shelf life of meat at a concentration of 0.25% (*w*/*v*) than at concentrations of 0.5 and 1% (*w*/*v*). Cui et al. [29] showed that *Morus alba* leaf extract significantly decreased TMC in chilled pork meat during storage compared to the control. Nowak et al. [14] demonstrated that sour cherry and blackcurrant leaf extracts extended the shelf life of vacuum packed pork sausages during 14 days of refrigerated storage. The extracts inhibited bacterial growth, decreasing TMC, TPC, LAB, *Pseudomonas* sp., *B. thermosphacta,* and *Enterobacteriaceae* counts.

### 2.2. Identification of Bacterial Communities in Meat Products by High-Throughput Sequencing of the 16S rRNA Gene

**Figure 2:**
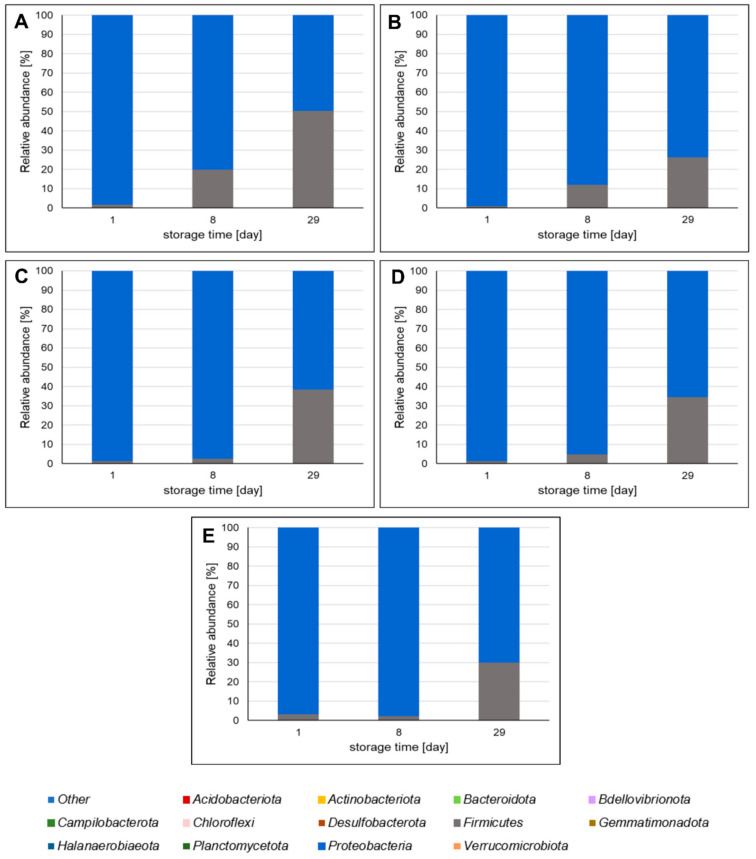
Bacterial phyla identified in pork sausages stored in MAP at 4 °C based on high-throughput sequencing of the 16S rRNA gene. The results are expressed as percentage share of each bacterial phylum in sausages. (**A**)—sausage with *Aronia melanocarpa* leaf extract; (**B**)—sausage with *Chaenomeles superba* leaf extract; (**C**)—sausage with *Cornus mas* leaf extract; (**D**)—sausage with curing salt containing sodium nitrate (III); (**E**)—control sausage with salt and spices only (without extract and nitrates (III)).

The bacterial communities in the different variants of pork meat products stored in MAP were identified using high-throughput sequencing on an Illumina platform. Samples of the sausages were selected for metagenomic analysis after 1, 8, and 29 days of storage. The results of our studies show that *A. melanocarpa*, *C. superba,* and *C. mas* leaf extracts influence microbial community in pork sausages. Figure 2 presents the bacterial phyla identified in the meat products. The predominant phyla in all sausage variants were *Firmicutes* and *Proteobacteria*. During refrigerated storage, the relative abundance of *Firmicutes* increased, whereas the relative abundance of *Proteobacteria* decreased. At the beginning of the storage period, *Proteobacteria* constituted 96.61–99.02% of OTUs (operational taxonomic units). However, by the end of the storage period, they represented 49.32–73.50% of OTUs, depending on the sausage variant. It is worth noting that the relative abundance of *Firmucutes* at the beginning of storage was only 0.95–3.09% of OTUs. However, after 29 days, it reached 26.42–50.64% of OTUs, depending on the sausage variant. The most significant change was observed in the case of the AMS sample. As well as *Firmicutes* and *Proteobacteria*, other bacterial phyla were detected in the meat products, including *Acidobaceriota, Actinobacteriota, Bacteroidota, Bdellovibrionota, Campilobacteriota, Chloroflexi, Desulfobacteriota, Gemmatimonadota, Halanaerobiaeota, Planctomycetota,* and *Verrucomicrobiota*.

**Figure 3:**
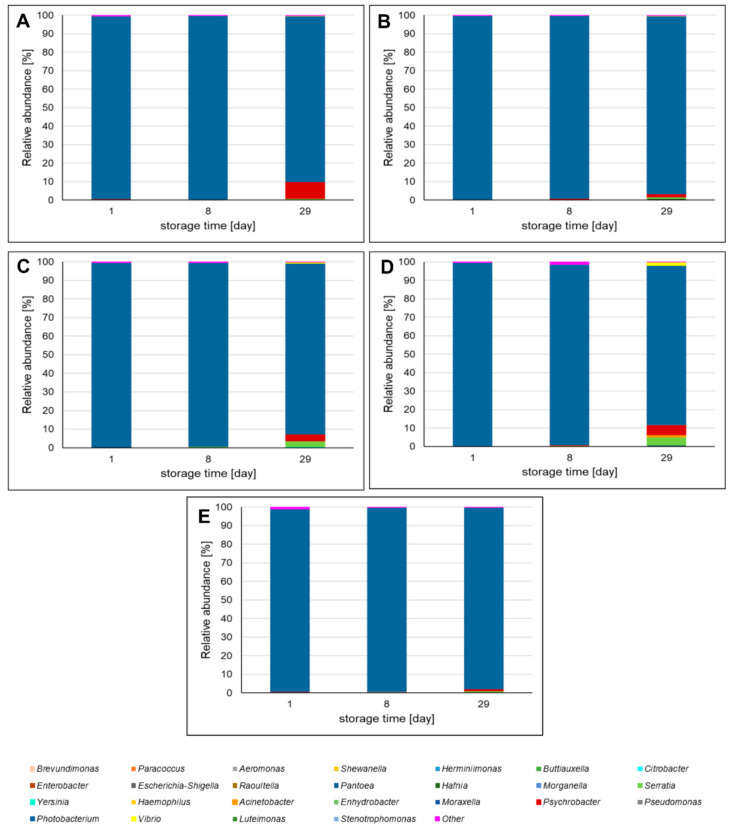
The most abundant genera among *Proteobacteria* phylum identified in pork sausages stored in MAP at 4 °C based on high-throughput sequencing of the 16S rRNA gene. The results are expressed as percentage share of each bacterial genius making up the total number of identified genera in each sausage sample among *Proteobacteria* phylum. Bacteria genera with percentage relative abundance >0.01% are shown. (**A**)—sausage with *Aronia melanocarpa* leaf extract; (**B**)—sausage with *Chaenomeles superba* leaf extract; (**C**)—sausage with *Cornus mas* leaf extract; (**D**)—sausage with curing salt containing sodium nitrate (III); (**E**)—control sausage with salt and spices only (without extract and nitrates (III)).

Figure 3 presents the most abundant genera among *Proteobacteria* phylum. The predominant genus was *Photobacterium* sp., which at the start of storage represented 98.19–99.54% of *Proteobacteria* OTUs, depending on the sausage variant. During storage, the amount of *Photobacterium* sp. decreased slightly, reaching 86.08–97.60% after 29 days. It was also observed that the relative abundance of *Psychrobacter* sp., *Serratia* sp., and *Vibrio* sp. increased during storage. In addition, the influence of extracts on bacteria diversity among *Proteobacteria* phylum was noticed. The smallest change in bacterial diversity was detected in the case of the SS sample. The relative abundance of *Psychrobacter* sp. and *Serratia* sp. was higher in samples containing leaf extracts and sodium nitrate (III), compared to the SS sample. *A. melanocarpa* had the greatest influence on *Psychrobacter* sp. Moreover, *C. mas* and *C. superba* leaf extracts as well as sodium nitrate (III) increased the relative abundance of *Vibrio* sp. This suggests that both the leaf extracts and nitrates (III) have inhibitory activity against *Photobacterium* sp. and/or can stimulate the growth of *Psychrobacter* sp., *Serratia* sp., and *Vibrio* sp.

**Figure 4:**
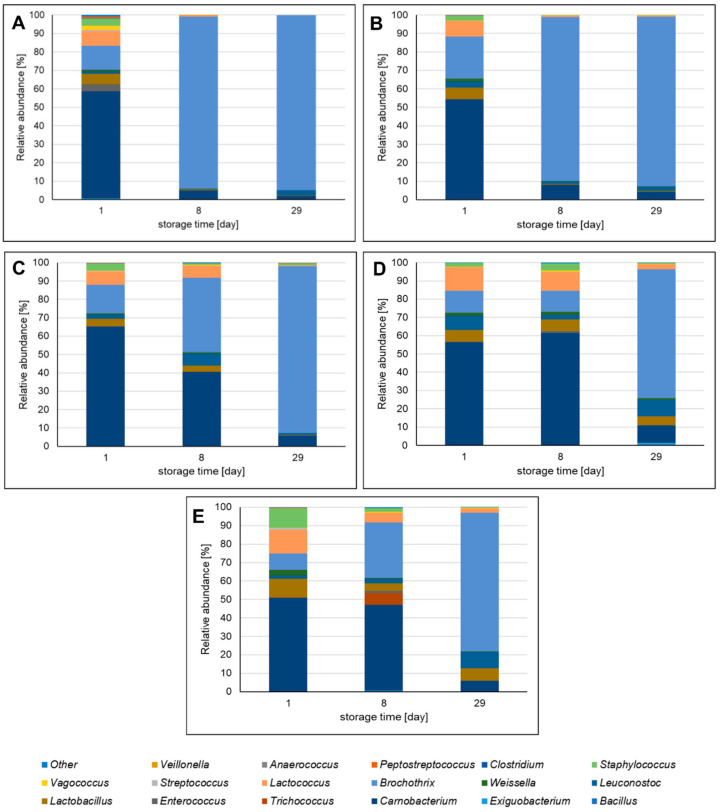
The most abundant genera among *Firmicutes* phylum identified in pork sausages stored in MAP at 4 °C based on high-throughput sequencing of the 16S rRNA gene. The results are expressed as the percentage share of each bacterial genius making up the total number of identified genera in each sausage sample among *Firmicutes* phylum. Bacteria genera with percentage relative abundance >0.01% are shown. (**A**)—sausage with *Aronia melanocarpa* leaf extract; (**B**)—sausage with *Chaenomeles superba* leaf extract; (**C**)—sausage with *Cornus mas* leaf extract; (**D**)—sausage with curing salt containing sodium nitrate (III); (**E**)—control sausage with salt and spices only (without extract and nitrates (III)).

At the start of storage, the *Firmicutes* phylum was mainly represented by *Carnobacterium* sp. (50.30–65.16% of *Firmicutes* OTUs) (Figure 4). However, during storage, *Brochothrix* sp. came to predominate, constituting after 29 days 70.29–94.65% of *Firmicutes* OTUs. During storage, the relative abundance of *Lactococcus* sp., *Lactobacillus* sp., and *Staphylococcus* sp. also decreased. It was noticed that the extracts influenced the diversity of *Firmicutes* phylum in pork sausages. In the AMS and ChS samples, and to a lesser degree in the CMS sample, the microbial community profile changed rapidly after 8 days of storage compared to the NS and SS samples. This suggests that leaf extracts inhibit the growth of *Carnobaterium* sp. and/or favor the growth of *Brochothrix* sp. The relative abundance of *Lactococcus* sp., *Lactobacillus* sp., and *Staphylococcus* sp. was lower in sausages with plant extracts than in NS and SS samples, which also suggests the inhibitory activity of leaf extracts. 

Our research showed that depending on the storage time, three bacteria genera generally predominated: *Photobacterium*, *Brochothrix*, and *Carnobacterium*. The high abundance of *Photobacterium* sp. in the pork sausages was unexpected. According to the literature, these bacteria do not usually constitute one of the predominant microflora in meat, including pork meat [1,2,3,4]. However, our results are in agreement with a study by Nieminen et al. [30], which also found that the bacterial communities in pork meat were composed predominantly of *Photobacterium* sp. and *Brochothrix* sp., as well as *Carnobacterium* sp., *Lactobacillus* sp., *Lactococcus* sp., and *Leuconostoc* sp. According to the same research, the relative abundance of *Photobacterium* sp. decreased during storage, while the relative abundance of *Brochothrix* sp. increased.

Since they occur mainly in seawater, sediment, or from interactions with marine animals, *Photobacterium* sp. are associated mainly with spoiled fish and seafood products [31,32]. However, several studies have noted the presence of these bacteria in meat products, including beef, chicken, turkey, and pork meat [30,33,34]. Hilgarth et al. [34] found *Photobacterium* sp. in 50% of tested meat samples, in quantities of up to 10^7^ CFU/g. They were isolated from 53% of the chicken samples, 22% of the pork meat samples, and 100% of the beef samples, as well as in 100% of the salmon samples. The main species of *Photobacterium* found in meat are *Photobacterium phosphoreum, Photobacterium carnosum,* and *Photobacterium iliopiscarium* [33].

*Photobacterium* sp. are Gram-negative bacteria belonging to the *Vibrioneceae* family. They are psychrotrophic or psychrophilic, depending on the species and strain. They are facultatively anaerobic bacteria and can occur in food products packed in different atmospheres, including in air, a vacuum, or MAP [33]. *Photobacterium* sp. are responsible for spoilage processes in meat. They are able to produce biogenic amines from amino acids, mainly histamine, tyramine, cadaverine, putrescine, and agmatine. They also synthesize ethanol, acetate, diacetyl, formate, lactate, and acetoin from pyruvate, which originates from carbohydrates. In the absence of carbohydrates, pyruvate can be obtained from citrate or glycerol, which are the constituents of lipids in meat. *Photobacterium* sp. can also use fat (triglycerides) as an energy source for growth. The metabolic pathways of *Photobacterium* sp. are similar in aerobic and anaerobic packaging atmospheres, such as CO_2_/O_2_ (30/70%) and CO_2_/N_2_ (30/70%) [35]. 

*Brochothrix* sp. and *Carnobacterium* sp. possess metabolic traits similar to *Photobacterium* sp. [35,36], which could explain the competition between these three genera observed in our studies. Previously, it was thought that *Brochothrix* sp. predominated in high-oxygen atmospheres, where they are able to use a wider range of substrates than in anaerobic atmospheres [37]. However, recent studies show that the methabolic pathways of *Brochothrix* sp. may be similar in both atmospheres [36]. In our study, the sausages were packed in anaerobic MAP (80% N_2_ and 20% CO_2_) and *Brochothrix* sp. predominated. *Brochothrix thermosphacta* are often detected in meat products. Nowak et al. [37] reported the presence of these bacteria in 130 out of 132 meat samples, in quantities of 10^1^–10^9^ CFU/g in meat and 10^2^–10^8^ CFU/g in meat products.

*Photobacterium* sp. need a complex medium with a high content of NaCl to grow [32]. In our studies, we used a standard medium (PCA), and these bacteria were not detected. High-throughput sequencing of 16S rRNA gene revealed that *Photobacterium* sp. was one of the predominant microflora in the pork meat sausages. Therefore, in future work, a culturing procedure for these bacteria will be established. Although *Photobacterium* sp. are not included in microbiological analysis of meat, studies show that they are one of the main groups of bacteria responsible for spoilage processes. Therefore, we recommend testing for *Photobacterium* sp. and *Brochothrix* sp. in standard microbiological analyses of meat.

### 2.3. Effect of Leaf Extracts on TBARS

**Figure 5:**
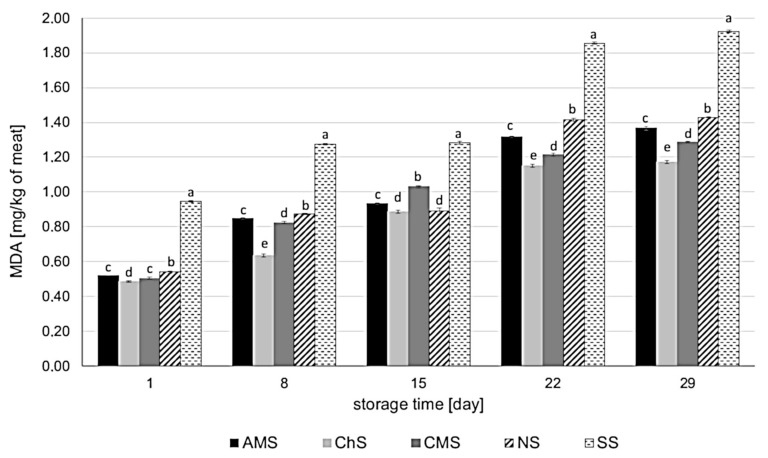
Effect of *Aronia melanocarpa, Chaenomeles superba*, and *Cornus mas* leaf extracts on lipid oxidation in pork sausages stored in MAP at 4 °C. The results are expressed as mean ± SD. a, b, c, d, e—statistically significant differences between the variants of sausages (*p* < 0.05); AMS—sausage with *Aronia melanocarpa* leaf extract (5% *v*/*w*); ChS—sausage with *Chaenomeles superba* leaf extract (5% *v*/*w*); CMS—sausage with *Cornus mas* leaf extract (5% *v*/*w*); NS—sausage with curing salt containing sodium nitrate (III); SS—control sausage with salt and spices only (without extract or nitrates (III).

Over 29 days of storage in MAP at 4 °C for 29 days, the MDA level increased in all sausage variants (Figure 5). The *A. melanocarpa, C. superba,* and *C. mas* leaf extracts significantly inhibited lipid oxidation. The highest level of MDA was observed in the case of the SS sample. The *C. superba* extract showed the strongest influence on lipid oxidation, followed by the *C. mas* and *A. melanocarpa* extracts. The MDA concentration in the sausages containing leaf extracts (AMS, ChS, and CMS) was even lower than in the NS sample, indicating stronger antioxidant properties compared to sodium nitrate (III). During refrigerated storage, the greatest increase in MDA level was detected in the SS sample (by 0.98 mg_MDA_/kg) followed by NS (by 0.89_MDA_ mg/kg), AMS (0.85 mg_MDA_/kg), CMS (0.78 mg_MDA_/kg), and ChS (by 0.69 mg_MDA_/kg). The effect of *A. melanocarpa, C. superba*, and *C. mas* leaf extracts on lipid oxidation is associated with the total phenolic content in the extracts and their antioxidant capacity, which had been determined in our previous study [24]. 

Lipid oxidation is one of the most important parameters determining the quality and shelf life of meat. It is responsible for off-flavors, unacceptable taste, discoloration, loss of nutritional value, the formation of toxic compounds, drip loss, and other alterations that can influence the perception and acceptance of meat by consumers [38]. It is extremely important to add antioxidant substances to meat, due to the fact that sodium chloride can reveal prooxidant properties and stimulate lipid oxidation. There are three possible mechanisms for the pro-oxidant activity of sodium chloride: (1) the disruption of cell membrane integrity, facilitating the access of oxidizing agents to lipid substrates; (2) the liberation of iron ions from molecules containing iron; (3) inhibiting the activity of antioxidant enzymes (e.g., catalase, glutathione peroxidase, superoxide dismutase) [39]. Other chloride salts, especially potassium chloride, can substitute sodium chloride and reduce its prooxidant effect [40,41]. However, due to their antioxidant, antibacterial activity, and many health benefits, natural plant extracts rich in polyphenols seem a more interesting alternative [9,11].

The antioxidant properties of plant extracts in meat and meat products are well documented in the literature. Plant extracts can effectively inhibit oxidative changes in meat ingredients, suppressing spoilage processes [9,11]. In a study by Biswas et al. [19], curry and mint leaf extracts reduced the MDA concentration in raw ground pork meat during 12 days of refrigerated storage. The curry leaf extract was more effective than both the mint leaf extract and sodium nitrate (III). Saleh et al. [13] reported that poultry meat treated with olive leaf extracts decreased TBARS values compared to the control, especially after 9, 12, and 15 days of storage. After 6 days, the control samples had a rancid flavor, whereas the samples treated with the extract had a normal flavor until the end of the storage period (15 days). According to Nowak et al. [14], sour cherry and blackcurrant water leaf extracts had an inhibitory effect on MDA generation in vacuum-packed pork sausages during 28 days of storage at 4 °C. The sausages were heat treated, cooked, and smoked, as in our study. The antioxidative efficiency was as follows: curing salt >blackcurrant leaf extract >sour cherry leaf extract >salt. Lavado et al. [15] showed that cork oak leaf extracts obtained using different solvents (water, ethanol, water: ethanol (1:1 *v*/*v*), and water: ethanol (3:7 *v*/*v*)) significantly inhibited lipid oxidation in cooked chicken breasts during 10 days of storage. Their antioxidant effect was similar to that of the chemical preservative butylated hydroxytoluene (BHT) at a concentration of 2% (*v*/*w*). No major differences between the types of solvents were observed. As in the studies by Nowak et al. [14] and Lavado et al. [15], we used water extracts and the sausages were heat treated. It is important to note that after thermal treatment of meat products, the extracts remained active and inhibited lipid oxidation.

### 2.4. Effect of Leaf Extracts on Drip Loss

Water-holding capacity is one of the most important quality characteristics of fresh meat. It influences the drip loss, technological quality, appearance, nutritional value, and sensory properties of meat products. During storage, the water-holding capacity of meat decreases and drip loss increases. This means that meat gradually loses its nutritional properties. As mentioned above, polyphenols can bind some substances in meat, such as proteins, carbohydrates, fiber, and fat [27,28], which can help prevent the loss of these substances during storage. Previous studies have only considered the dietary supplementation of animal feed with plant extracts [16,42]. Shen et al. [16] demonstrated that the drip loss from chicken breast meat after 24 h postmortem decreased when the animals were fed a diet supplemented with bamboo leaf extract. Ao and Kim [42] report that supplementation of feed with grape seed extract decreased the drip loss from duck meat on days 3 and 5. 

**Figure 6:**
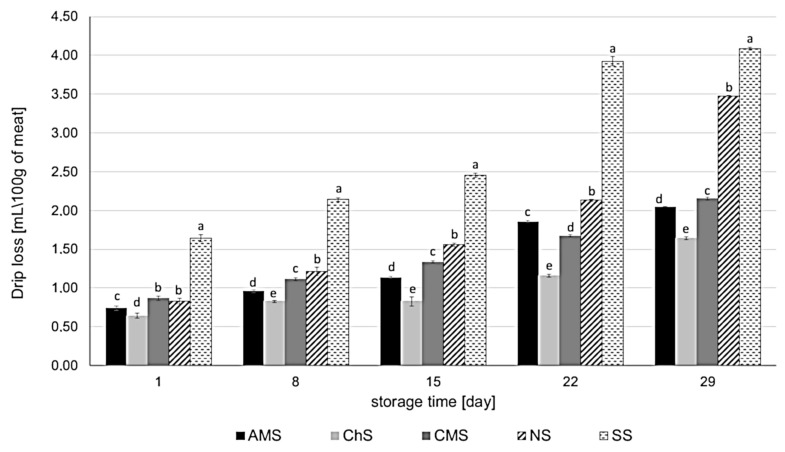
Effect of *Aronia melanocarpa, Chaenomeles superba,* and *Cornus mas* leaf extracts on drip loss in pork sausages stored in MAP at 4 °C. The results are expressed as mean ± SD. a, b, c, d, e—statistically significant differences between the variants of sausages (*p* < 0.05); AMS—sausage with *Aronia melanocarpa* leaf extract (5% *v*/*w*); ChS—sausage with *Chaenomeles superba* leaf extract (5% *v*/*w*); CMS—sausage with *Cornus mas* leaf extract (5% *v*/*w*); NS—sausage with curing salt containing sodium nitrate (III); SS—control sausage with salt and spices only (without extract and nitrates (III)).

In the present research, we found that the studied plant extracts significantly improved the water-holding capacity of pork sausages during 29 days of refrigerated storage (4 °C) in MAP (Figure 6). The extracts showed an even stronger influence on drip loss than sodium nitrate (III). During storage, the drip loss of the variants of pork sausages studied was as follows: SS > NS > AMS ~ CMS > ChS. After 29 days of storage, the drip loss from the sausages increased by 2.64 mL/100 g (NS), 2.44 mL/100 g (SS), 1.31 mL/100 g (AMS), 1.29 mL/100 g (CMS), and 1.00 mL/100 g (ChS), compared to day 1. After 29 days, a significant increase in drip loss was observed in the case of the NS sample, whereas the samples with extracts still revealed strong protective ability. Water-holding capacity was associated with total phenolic content. The *C. superba* extract contained the highest amount of phenolic compounds, followed by the *C. mas* and *A. melanocarpa* extracts [24].

### 2.5. Effect of Leaf Extracts on Color Parameters

Table 2 shows the color parameters of the pork sausages containing *A. melanocarpa, C. superba,* and *C. mas* leaf extracts during refrigerated storage in MAP. The lightness was generally stable in all the sausage variants during 29 days of storage. The most important color parameter of meat is redness (a*), which is a characteristic associated with meat products. The highest a* values were detected for the NS sample. However, the extracts significantly improved the redness of the sausages compared to the SS sample over 29 days of storage. The greatest influence was observed in the case of the AMS sample, followed by ChS and CMS. This was confirmed by the total color difference, calculated in comparison to the NS sample (ΔE_NS_). After 8–22 days of storage, the ΔE_NS_ of the AMS sample was the lowest. We also observed that the redness of the AMS, ChS, CMS, and NS samples increased significantly after 8 days and remained stable during the rest of the storage period. The yellowness of the AMS, ChS, NS, and SS samples did not change significantly during storage. In the case of the AMS sample, a significant decrease in b* values was observed over the whole period of storage.

Ramírez-Rojo et al. [43] reported that mesquite leaf extracts affected the color parameters of pork patties. Initially, the a* values were lower than in the control sample (by 27.8%), and the b* values were higher (by 42.1%) However, after 10 days of storage, the patties treated with extracts displayed the highest redness and the highest yellowness compared to the control. In a study by Nowak et al. [14], sour cherry and blackcurrant leaf extract were not found to exert a strong influence on the color changes in vacuum-packed pork sausages. Only the blackcurrant leaf extract slightly improved the redness of the meat products compared to the control. Tran et al. [44] report that guava leaf extracts improved the redness of pork sausages, which was comparable to a sample with BHT. However, after 14 days, higher concentrations of the extract showed a prooxidant effect on meat color, which could be detrimental. According to Zhang et al. [45], cauliflower leaf extract increased a* values and decreased L* values in pork patties compared to the control.

### 2.6. Sensory Evaluation of Meat Products

Table 3 shows the sensory properties of the pork sausages stored in MAP at 4 °C. Generally, the extracts did not have a negative impact on the sensory properties of the meat products up to 15 days. The sensory parameters were stable during 8 days of storage. After 15 days, a slight change in odor was observed in the AMS, ChS CMS, and SS samples. The leaf extracts, especially *A. melanocarpa* and *C. superba*, which were red in color, improved the color of the sausages, which was between the SS and NS samples. No negative effect was detected in terms of taste. The texture of the sausages with *A. melanocarpa* extract was similar to that of the NS sample. However, the *C. mas* extract had a negative influence on texture. After 22 days, the texture of the CMS samples was uneven, and green traces were observed. The green traces could be due to the release of the extract, which had yellow-green color, or to the activity of microorganisms. This negative change was not observed earlier in the storage period.

According to the literature, meat products enriched with plant extracts containing polyphenols are generally acceptable to consumers, and the extracts do not have a negative impact on their sensory characteristics. In a study by Nowak et al. [14], cherry and blackcurrant leaf extracts were not found to have a negative impact of on the sensory properties of pork sausages, which remained acceptable throughout 14 days of storage. Cui et al. [29] report that *Morus alba* leaf extract extended the shelf life of pork meat, based on sensory analysis. In contrast to polyphenolic extracts, essential oils can reduce organoleptic acceptability in terms of taste and odor [46].

## 3. Materials and Methods

### 3.1. Research Material

The research material consisted of *Aronia melanocarpa, Chaenomeles superba,* and *Cornus mas* water leaf extracts. The material was exactly the same as in our previous studies, with the same phenolic content. The total phenolic contents of each extract determined using the Folin–Ciocalteu method were as follows: 861.6 µg/mL (*A. melanocarpa*); 3110.6 µg/mL (*C. superba*); 1867.7 µg/mL (*C. mas*) [24].

### 3.2. Preparation of Sausages

Three classes of pork meat with different fat contents were used to prepare sausages: class 1 (lean, up to 15% fat), class 2a (medium fat, 16–20%), and class 2b (fat, 21–45%). The sausages were manufactured at 4 °C. The meat was cut into small cubes and mixed in the following proportions: 10% lean; 60% medium fat; 30% fat. Water, NaCl, spices (pepper and garlic), curing salt, and plant extracts were mixed thoroughly into the meat, according to the proportions shown in Table 4. The following variants of sausages were prepared: AMS—sausages with the addition of *A. melanocarpa* leaf extract; ChS—sausages with the addition of *C. superba* leaf extract; CMS—sausages with the addition of *C. mas* leaf extract; NS—sausages with the addition of a curing mixture containing sodium nitrate (III); SS—control sausages without extract. The meat samples were stored and cured at 4 ± 2 °C for 24 h; then, they were minced using a PM-70 Mincer (MAINCA, Granollers, Spain). The minced meat was stuffed into natural casings using an EC-12 filling machine (MAINCA, Granollers, Spain). Next, the sausages were transported to the Marczak meat processing plant (Dobrzelow, Poland. They were cooked in a steam smokehouse at a temperature of 72 °C inside the sausage and then smoked using traditional alder and beech wood smoke. After thermal processing, the sausages were cooled, packed in a modified atmosphere (80% N_2_ and 20% CO_2_), and stored at 4 °C for 29 days. 

### 3.3. Microbiological Counts

Microbiological analysis of the sausages was performed after 1, 4, 8, 11, 15, 22, and 29 days of refrigerated storage at 4 °C. The samples were prepared according to ISO 6887-2-2017. The total psychrotrophic count (TPC) was determined on Plate Count Agar (PCA) (Merck, Darmstadt, Germany) following incubation at 6 °C for 6 days. The lactic acid bacteria (LAB) were counted on Man Rogosa Sharpe Agar (MRS) (Merck, Darmstadt, Germany) following incubation for 72 h at 30 °C. The quantity of *Enterobacteriaceae* was determined on Violet Red Bile Dextrose Agar (VRBD) (Merck, Darmstadt, Germany) following incubation at 30 °C for 24 h. The lowest detection limit of the applied enumeration techniques is 10 CFU/g.

### 3.4. DNA Extraction, PCR Amplification, and Sequencing

The total DNA was extracted directly from the meat (0.5 g) using a DNeasy PowerFood Microbial Kit (Qiagen), according to the manufacturer’s instructions. DNA concentrations were assessed using a Qubit 2.0 Fluorometer (Invitrogen/Life Technologies, Carlsbad, CA, USA).

For amplification of the V3-V4 region of the 16S rRNA gene, the universal primer pair 341F/785R was used. PCR reactions were carried out using the Q5 Hot Start High Fidelity 2× Master Mix (New England BioLabs Inc., Ipswich, MA, USA) under thermal conditions: initial denaturation 98 °C for 30 s, followed by 25 cycles of denaturation 98 °C for 10 s, annealing at 55 °C for 30 s, extension 72 °C for 30 s, and final extension for 2 min at 72 °C. 

DNA libraries were constructed using a Nextera Index Kit (ThermoFisher Scientific, USA) following the protocol provided by manufacturer. High-throughput sequencing of 16S rRNA libraries were performed on the Illumina MiSeq platform by Genomed S.A. (Warsaw, Poland).

Preliminary data analysis was carried out using a MiSeq apparatus with MiSeq Reporter (MSR) v 2.6 software (Illumina, Inc., San Diego, CA, USA). Each sample was demultiplexed, and fastq files containing raw reads were generated. Bioinformatic analysis to classify the readings was carried out using QIIME 2 software based on the SILVA v 138 database. The procedure consisted of the following steps: removal of adapter sequences; evaluation of the quality of the readings and removal of low-quality sequences connecting paired sequences; clustering based on the selected database of reference sequences and removal of sequence chimeras [47,48,49]. The sequences were clustered using the uclust algorithm [50]. Operational Taxonomic Units (OTUs) were assigned to taxa to the selected database of reference sequences. 

### 3.5. Determination of TBARS 

Lipid stability was evaluated according to the method proposed by Targladis et al. [51], with some modifications. All sausage variants were analyzed after 1, 8, 15, 22, and 29 days of refrigerated storage. The experiments were conducted in triplicate. The sausage samples were minced using a Zelmer 686 meat mincer (Zelmer S.A., Rzeszow, Poland). A 5 g sample with 75 mL of distilled water and 7 mL of 3M HCl was transferred to a distillation system. About 20 mL of the distillate was collected and 3 mL of 0.02 M thiobarbituric acid (TBA) was added. The reaction mixture was heated in a boiling bath for 35 min and cooled to room temperature. The absorbance was measured at 530 nm against distilled water using a T60V spectrophotometer (PG Instruments, Leicestershire, United Kingdom). The amounts of thiobarbituric acid reactive substances (TBARS) were determined according to the standard curve (R^2^ = 0.98) and expressed as mg malondialdehyde (MDA)/kg of meat.

### 3.6. Drip Loss

Drip loss was determined using the filter paper press method described by Grau and Hamm [52], with some modifications. The sausage samples were minced. A total of 0.5 g of each sample was placed on the paper filter between two cover glasses under a pressure of 200 g for 10 min. Using a planimeter, the difference (RZ) between the wet area on the filter paper (T) and the area of the pressed meat (M) was determined. The amount of water that leaked from the meat sample was calculated according to the calibration curve (R^2^ = 0.99).

### 3.7. Instrumental Color Measurement

Color parameters were measured using a CR-400 Chroma Meter (Minolta Ltd., Milton Keynes, UK). The meat samples were minced and measurements were performed directly on the surface of the meat. The color parameters were determined using the CIEL*a*b* system. The results were expressed as lightness (L*), redness (a*) and yellowness (b*). The color difference (ΔE*) was calculated as follows:

$${\Delta E}^{2}=\sqrt{{\left({\Delta a}^{*}\right)}^{2}+{\left({\Delta b}^{*}\right)}^{2}+\left({\Delta L}^{*}{)}^{2}\right)}.$$

### 3.8. Sensory Analysis

A trained panel of 10 judges evaluated the sensory properties of the pork sausages at selected points during refrigerated storage. The samples were assessed in terms of taste, color, odor, and texture. Each attribute was scored on a 5-point descriptive scale, where 5 is excellent and 1 is entirely unacceptable. 

### 3.9. Statistical and Chemometric Analysis

Mean values and standard deviations were calculated using Microsoft Excel 2013 software. The Tukey Honestly Significant Difference (HSD) test was performed using R 3.4.0 software (R Core Team, Vienna, Austria) (*p* < 0.05). Principal Component Analysis (PCA) was performed using Statistica 13 software (StatSoft, Poland, Kraków).

## 4. Conclusions

In this study, *A. melanocarpa*, *C. superba,* and *C. mas* leaf extracts were added to pork sausages at a concentration of 5% (*v*/*w*), corresponding to a phenolic content of 43.08 mg/kg of meat for *A. melanocarpa*, 155.53 mg/kg of meat for *C. superba,* and 93.38 mg/kg of meat for *C. mas*. The extracts improved the microbial quality of meat products during refrigerated storage in MAP but to a lesser extent than sodium nitrate (III). The extracts decreased *Enterobacteriaceae* and LAB counts with *A. melanocarpa* showing the greatest influence. Two bacterial phyla predominated in pork sausages, *Proteobacteria* and *Firmicutes,* especially the genera *Photobacterium* sp., *Brochothrix* sp., and *Carnobacterium* sp. Therefore, we recommend testing for *Brochothrix* sp. and *Photobaterium* sp. in routine microbiological analysis of meat and meat products. The leaf extracts also influenced microbial community of meat products decreasing or increasing bacterial relative abundance (OTUs). The leaf extracts inhibited lipid oxidation and improved the water-holding capacity of the meat (*C. superba* > *C. mas* > *A. melanocarpa*). The extracts (especially *A. melanocarpa* and *C. superba*) increased the redness of the sausages, improving the visual attributes of the meat products. The addition of leaf extracts to the meat products did not have a negative effect on their sensory properties up to 15 days of storage.

## Figures and Tables

**Table 1:** Effect of *Aronia melanocarpa, Chaenomeles superba* and *Cornus mas* leaf extracts on microbial counts in pork sausages stored in MAP at 4 °C (mean ± SD).

	LOG(CFU/g)						
Storage Time (Day)	1	4	8	11	15	22	29
	TPC						
AMS	2.62 ± 0.11 ^c^	3.79 ± 0.05 ^c^	6.33 ± 0.08 ^b^	6.57 ± 0.11 ^c^	7.14 ± 0.08 ^c^	7.54 ± 0.04 ^b^	7.66 ± 0.17 ^b^
ChS	3.89 ± 0.10 ^a^	4.07 ± 0.06 ^b^	6.20 ± 0.08 ^b^	7.33 ± 0.03 ^a^	8.30 ± 0.04 ^a^	7.89 ± 0.08 ^a^	8.15 ± 0.11 ^a^
CMS	3.85 ± 0.06 ^a^	4.48 ± 0.02 ^a^	5.82 ± 0.11 ^c^	6.25 ± 0.06 ^d^	6.90 ± 0.07 ^d^	7.22 ± 0.06 ^c^	8.13 ± 0.08 ^a^
NS	3.33 ± 0.06 ^b^	3.72 ± 0.04 ^c^	4.21 ± 0.13 ^d^	4.98 ± 0.27 ^e^	6.39 ± 0.34 ^e^	6.67 ± 0.19 ^d^	7.49 ± 0.10 ^b^
SS	3.91 ± 0.08 ^a^	4.46 ± 0.05 ^a^	6.81 ± 0.11 ^a^	6.98 ± 0.06 ^b^	7.50 ± 0.13 ^b^	8.05 ± 0.14 ^a^	7.93 ± 0.09 ^a^
	***Enterobacteriaceae***						
AMS	1.65 ± 0.08 ^a.b^	1.00 ± 0.02 ^c^	2.62 ± 0.26 ^b^	2.50 ± 0.18 ^e^	3.65 ± 0.07 ^b^	4.69 ± 0.12 ^b.c^	5.13 ± 0.02 ^c^
ChS	1.66 ± 0.22 ^a.b^	1.63 ± 0.16 ^b^	2.81 ± 0.09 ^b^	3.77 ± 0.10 ^c^	4.57 ± 0.15 ^a^	4.61 ± 0.12 ^c^	6.23 ± 0.21 ^a^
CMS	1.74 ± 0.06 ^a^	1.70 ± 0.05 ^b^	2.72 ± 0.20 ^b^	4.18 ± 0.16 ^b^	4.71 ± 0.10 ^a^	5.61 ± 0.10 ^a^	6.24 ± 0.14 ^a^
NS	1.45 ± 0.21 ^b^	1.70 ± 0.07 ^b^	1.78 ± 0.06 ^c^	2.88 ± 0.05 ^d^	3.29 ± 0.31 ^b^	4.30 ± 0.18 ^d^	5.30 ± 0.18 ^b.c^
SS	1.65 ± 0.07 ^a.b^	2.32 ± 0.15 ^a^	3.42 ± 0.16 ^a^	4.47 ± 0.21 ^a^	4.64 ± 0.10 ^a^	4.96 ± 0.18 ^b^	5.41 ± 0.17 ^b^
	**LAB**						
AMS	1.45 ± 0.21 ^c^	2.00 ± 0.13 ^c^	3.31 ± 0.18 ^b^	3.64 ± 0.13 ^c^	4.89 ± 0.05 ^c^	5.71 ± 0.08 ^c^	6.17 ± 0.02 ^c^
ChS	2.00 ± 0.13 ^b^	2.52 ± 0.20 ^b^	2.82 ± 0.09 ^c^	4.86 ± 0.09 ^a^	5.58 ± 0.03 ^b^	6.19 ± 0.14 ^b^	6.32 ± 0.01 ^b^
CMS	2.40 ± 0.12 ^a^	2.86 ± 0.08 ^a^	3.34 ± 0.05 ^b^	3.55 ± 0.20 ^c^	3.58 ± 0.23 ^d^	6.45 ± 0.02 ^a^	6.15 ± 0.04 ^c^
NS	1.48 ± 0.18 ^c^	1.93 ± 0.04 ^c^	3.42 ± 0.06 ^b^	4.69 ± 0.14 ^a.b^	5.65 ± 0.05 ^b^	5.18 ± 0.13 ^d^	6.14 ± 0.13 ^c^
SS	2.33 ± 0.18 ^a^	2.75 ± 0.06 ^a.b^	4.17 ± 0.18 ^a^	4.65 ± 0.05 ^b^	5.94 ± 0.09 ^a^	6.04 ± 0.07 ^b^	6.74 ± 0.09 ^a^

^a,b,c,d,e^—statistically significant differences between the variants of sausages (*p* < 0.05): AMS—sausage with *Aronia melanocarpa* leaf extract (5% *v*/*w*); ChS—sausage with *Chaenomeles superba* leaf extract (5% *v*/*w*); CMS—sausage with *Cornus mas* leaf extract (5% *v*/*w*); NS—sausage with curing salt containing sodium nitrate (III); SS—control sausage with salt and spices only (without extract and nitrates (III)); TPC—total psychrotrophic count; LAB—lactic acid bacteria.

**Table 2:** Effect of *Aronia melanocarpa, Chaenomeles superba,* and *Cornus mas* leaf extracts on color parameters (Lightness L*, redness a*, yellowness b*) of pork sausages stored in MAP at 4 °C (mean ± SD).

Storage Time [Day]	1	8	15	22	29
Lightness L*					
AMS	63.90 ± 0.52 ^b,c A^	60.50 ± 0.26 ^c B^	63.24 ± 0.45 ^a,b A^	63.18 ± 0.27 ^a A^	64.14 ± 0.44 ^a A^
ChS	63.45 ± 0.13 ^c A,B,C^	62.10 ± 0.35 ^b B,C^	63.98 ± 0.30 ^b A^	62.06 ± 0.37 ^b C^	63.78 ± 0.33 ^a,b AB^
CMS	65.02 ± 0.93 ^a A^	62.70 ± 0.46 ^b A,B,C^	60.69 ± 0.36 ^b B,C^	59.78 ± 0.25 ^c C^	63.55 ± 0.33 ^a,b A,B^
NS	64.63 ± 0.29 ^a,b A^	61.13 ± 0.42 ^c A^	62.58 ± 0.26 ^a,b B^	63.15 ± 0.49 ^a A^	63.25 ± 0.51 ^b A^
SS	64.85 ± 0.51 ^a,b A,B^	65.47 ± 0.44 ^a A^	64.50 ± 0.33 ^a A,B^	62.93 ± 0.26 ^a C^	63.66 ± 0.07 ^a,b B,C^
Redness a*					
AMS	4.81 ± 0.23 ^c B^	8.14 ± 0.24 ^b A^	8.15 ± 0.09 ^b A^	8.08 ± 0.08 ^b A^	8.09 ± 0.14 ^b A^
ChS	5.93 ± 0.42 ^b B^	7.41 ± 0.18 ^c A^	8.12 ± 0.06 ^b A^	8.06 ± 0.13 ^b A^	7.29 ± 0.18 ^c A^
CMS	6.14 ± 0.30 ^a,b B^	7.70 ± 0.17 ^c A^	7.63 ± 0.10 ^c A^	7.12 ± 0.05 ^c A,B^	7.58 ± 0.05 ^c A^
NS	6.60 ± 0.20 ^a B^	11.01 ± 0.24 ^a A^	9.91 ± 0.13 ^a A^	9.08 ± 0.13 ^a A,B^	9.98 ± 0.21 ^a A^
SS	4.55 ± 0.28 ^c C^	5.52 ± 0.19 ^d B^	5.69 ± 0.03 ^d B^	6.59 ± 0.13 ^d A^	6.57 ± 0.39 ^d A^
Yellowness b*					
AMS	13.93 ± 0.19 ^a A^	13.57 ± 0.28 ^a A,B^	13.94 ± 0.07 ^a A^	13.00 ± 0.03 ^b B^	11.77 ± 0.10 ^d C^
ChS	12.14 ± 0.11 ^c C^	12.69 ± 0.03 ^b A,B^	12.52 ± 0.05 ^c A,B^	12.36 ± 0.02 ^d B,C^	12.73 ± 0.01 ^b A^
CMS	12.07 ± 0.30 ^c A^	11.22 ± 0.02 ^c B^	12.23 ± 0.14 ^d A^	12.55 ± 0.05 ^c A^	12.26 ± 0.10 ^c A^
NS	13.00 ± 0.11 ^b A,B^	12.49 ± 0.03 ^b B^	13.81 ± 0.06 ^a A^	13.03 ± 0.05 ^b A,B^	13.45 ± 0.30 ^a A^
SS	13.28 ± 0.18 ^b A^	13.93 ± 0.58 ^a A^	13.44 ± 0.06 ^b A^	13.52 ± 0.14 ^a A^	13.36 ± 0.01 ^a A^
**ΔE_NS_**					
AMS	2.21 ± 0.13 ^a B^	3.14 ± 0.35 ^c A^	1.91 ± 0.27 ^d B^	1.02 ± 0.11 ^d C^	2.71 ± 0.20 ^b A^
ChS	1.66 ± 0.16 ^b C^	3.74 ± 0.27 ^b,c A^	2.63 ± 0.18 ^c B^	1.67 ± 0.11 ^c C^	2.84 ± 0.24 ^b B^
CMS	1.44 ± 0.16 ^b D^	3.89 ± 0.30 ^b A^	3.36 ± 0.18 ^b B^	3.93 ± 0.20 ^a A^	2.71 ± 0.06 ^b C^
SS	2.14 ± 0.28 ^a D^	7.16 ± 0.45 ^a A^	4.65 ± 0.16 ^a B^	2.56 ± 0.15 ^b D^	3.44 ± 0.39 ^a C^

a, b, c, d—statistically significant differences between the sausage variants (in columns) (*p* < 0.05); A,B,C,D—statistically significant differences during storage (in rows) (*p* < 0.05); AMS—sausage with *Aronia melanocarpa* leaf extract (5% *v*/*w*); ChS—sausage with *Chaenomeles superba* leaf extract (5% *v*/*w*); CMS—sausage with *Cornus mas* leaf extract (5% *v*/*w*); NS—sausage with curing salt containing sodium nitrate (III); SS—control sausage with salt and spices only (without extract and nitrates (III)); ΔE_NS_—total color difference in comparison with the NS sample.

**Table 3:** Effect of *Aronia melanocarpa, Chaenomeles superba*, and *Cornus mas* leaf extracts on the sensory properties of pork sausages stored in MAP at 4 °C.

Storage Time [Day]	1	8	15	22	29	1	8	15	22	29
Sausage variant	Color (1–5)					Taste (1–5)				
AMS	4.5	4.5	4.5	4	4	5	5	n.t.	n.t.	n.t.
ChS	4.5	4.5	4.5	4	4	5	5	n.t.	n.t.	n.t.
CMS	4	4	4	3.5	3.5	5	5	n.t.	n.t.	n.t.
NS	5	5	5	4	4.5	5	5	n.t.	n.t.	n.t.
SS	3	3	3	2.5	2	5	5	n.t.	n.t.	n.t.
Sausage variant	Odor (1–5)					Texture (1–5)				
AMS	5	5	4.5	4	4	5	5	5	5	4.5
ChS	5	5	4.5	3.5	3	5	5	5	4.5	4
CMS	5	5	4.5	3.5	3	5	5	5	3	3
NS	5	5	5	4	4	5	5	5	5	4.5
SS	5	5	4	3	2.5	5	5	5	4	4

n.t.—not tested; AMS—sausage with *Aronia melanocarpa* leaf extract (5% *v*/*w*); ChS—sausage with *Chaenomeles superba* leaf extract (5% *v*/*w*); CMS—sausage with *Cornus mas* leaf extract (5% *v*/*w*); NS—sausage with curing salt containing sodium nitrate (III); SS—control sausage with salt and spices only (without extract and nitrates (III).

**Table 4:** Content of additives in the sausage variants.

Sausage Variant	Water	Plant Extract	NaCl	Curing Salt Containing Sodium Nitrate (III)	Pepper	Garlic
	mL/100g of Meat		mg/100g of Meat			
AMS	20	**5**	**1.8**	-	0.2	0.4
ChS	20	5	1.8	-	0.2	0.4
CMS	20	5	1.8	-	0.2	0.4
NS	20		-	1.8	0.2	0.4
SS	20	-	1.8	-	0.2	0.4

AMS—sausage with *Aronia melanocarpa* leaf extract; ChS—sausage with *Chaenomeles superba* leaf extract; CMS—sausage with *Cornus mas* leaf extract; NS—sausage with curing salt containing sodium nitrate (III); SS—control sausage with salt and spices only (without extract and nitrates (III)).

## Data Availability

Data is contained within the article.

## Sample Availability

Samples of the compounds are not available from the authors.
